# From Bench to Bedside: Implications of Lipid Nanoparticle Carrier Reactogenicity for Advancing Nucleic Acid Therapeutics

**DOI:** 10.3390/ph16081088

**Published:** 2023-07-31

**Authors:** Tetiana Korzun, Abraham S. Moses, Parham Diba, Ariana L. Sattler, Olena R. Taratula, Gaurav Sahay, Oleh Taratula, Daniel L. Marks

**Affiliations:** 1 Department of Pharmaceutical Sciences, College of Pharmacy, Oregon State University, 2730 S Moody Avenue, Portland, OR 97201, USA; korzun@ohsu.edu (T.K.);; 2 Department of Biomedical Engineering, Oregon Health & Science University, 3303 SW Bond Avenue, Portland, OR 97239, USA; 3 Medical Scientist Training Program, Oregon Health & Science University, 3181 SW Sam Jackson Park Road, Portland, OR 97239, USA; 4 Papé Family Pediatric Research Institute, Oregon Health & Science University, 3181 SW Sam Jackson Park Rd, Portland, OR 97239, USA; 5 Knight Cancer Institute, Oregon Health & Science University, 2720 S Moody Avenue, Portland, OR 97201, USA; 6 Brenden-Colson Center for Pancreatic Care, Oregon Health & Science University, 2730 S Moody Avenue, Portland, OR 97201, USA

**Keywords:** empty lipid nanoparticles, reactogenicity, xenobiotics, ionizable lipids

## Abstract

In biomedical applications, nanomaterial-based delivery vehicles, such as lipid nanoparticles, have emerged as promising instruments for improving the solubility, stability, and encapsulation of various payloads. This article provides a formal review focusing on the reactogenicity of empty lipid nanoparticles used as delivery vehicles, specifically emphasizing their application in mRNA-based therapies. Reactogenicity refers to the adverse immune responses triggered by xenobiotics, including administered lipid nanoparticles, which can lead to undesirable therapeutic outcomes. The key components of lipid nanoparticles, which include ionizable lipids and PEG-lipids, have been identified as significant contributors to their reactogenicity. Therefore, understanding the relationship between lipid nanoparticles, their structural constituents, cytokine production, and resultant reactogenic outcomes is essential to ensure the safe and effective application of lipid nanoparticles in mRNA-based therapies. Although efforts have been made to minimize these adverse reactions, further research and standardization are imperative. By closely monitoring cytokine profiles and assessing reactogenic manifestations through preclinical and clinical studies, researchers can gain valuable insights into the reactogenic effects of lipid nanoparticles and develop strategies to mitigate undesirable reactions. This comprehensive review underscores the importance of investigating lipid nanoparticle reactogenicity and its implications for the development of mRNA–lipid nanoparticle therapeutics in various applications beyond vaccine development.

## 1. Introduction

Nanomaterial-based delivery vehicles are often praised for their ability to encapsulate both hydrophobic and hydrophilic payloads, which include small molecule drugs, imaging agents, and nucleic acids, resulting in their enhanced solubility, extended circulation time, and delayed degradation in the systemic circulation [1]. These properties make nanomedicines an attractive alternative to traditional therapeutic and diagnostic modalities, leading to their extensive use clinically and pre-clinically in various biomedical applications [2]. Extensive research was conducted on the efficacy of lipid nanoparticles (LNPs) for the delivery of messenger RNA (mRNA) as a vaccination tool for the prevention of COVID-19 during the recent pandemic [3,4,5,6,7]. The potential applications of mRNA–LNPs for non-infectious diseases, including those necessitating protein supplementation or replacement therapies, were significantly enhanced by the progress made in this field [8]. These therapeutic interventions apply to a range of disorders, encompassing those linked to chronic inflammation, such as cancer, autoimmune diseases, cardiovascular disorders, diabetes, and other conditions. However, caution must be exercised when considering already reactogenic xenobiotic substances in the presence of pre-existing inflammation. Such a combination is likely to aggravate the underlying inflammation, hamper therapeutic mRNA translation, and interfere with the repeated administration of mRNA therapeutics.

Studies on the reactogenicity of mRNA–LNP formulations were conducted by leveraging the abundance of data derived from clinical trials of COVID-19 vaccines. Systemic and local reactogenic symptoms are known to occur after the administration of COVID-19 mRNA vaccines, including formulations developed by Pfizer-BioNTech and Moderna [4,9]. The observed side effects include fever, muscle aches, headaches, fatigue, chills, and pain at the injection site [3,6,7]. These symptoms are usually mild to moderate in severity and are more common after the second dose of the vaccine [10]. Individuals with underlying chronic inflammation may be at an increased risk of experiencing severe side effects from mRNA–LNP-based therapies and may also have a reduced response to the treatment itself [11]. Furthermore, as shown by COVID-19 vaccine reactogenicity studies, overactivation of immune responses triggered by mRNA–LNP formulations could lead to new-onset autoimmune disorders such as autoimmune liver diseases, Guillain–Barré syndrome, immune thrombotic thrombocytopenia, IgA nephropathy, rheumatoid arthritis, systemic lupus erythematosus, and others [11,12,13]. Despite the reported unfavorable outcomes, research demonstrates that the manifestation of vaccination side effects is linked to a more robust immune reaction and enhanced protection against COVID-19 [14,15]. However, to guarantee the safety and effectiveness of LNP carrier species for therapeutic applications beyond vaccines and infectious disease prevention, especially when immune activation is unnecessary, a thorough investigation of their toxicity and reactogenicity is indispensable during the formulation development and optimization stages.

The influence of multiple pro-reactogenic elements of mRNA–LNP formulations, such as ionizable lipids, the polyethylene glycol (PEG) coating, and mRNA cargo, should be studied with great caution. Extensive investigation concerning mRNA’s reactogenicity, stability, and translatability creates a favorable environment for the current applications of modified mRNA in various therapeutic settings [16,17,18]. For LNP reactogenicity research, the current focus is now centered on investigating the reactogenic adjuvanticity of LNP formulations to enhance the immunogenicity of mRNA–LNP vaccine formulations [19]. Although a thorough investigation of the LNP carrier’s reactogenicity is yet to be accomplished, the current data raise important questions revolving around LNP-associated side effects. For instance, the use of a greater mRNA–LNP dose in the mRNA-1273 vaccine and different ionizable lipids used in the formulation are potential explanations for the increased reactogenicity of mRNA-1273 compared with BNT162b formulations in the Moderna and Pfizer-BioNTech COVID-19 vaccines, respectively [20,21]. Therefore, further research and standardization in the area of reactogenic manifestations and their association with the LNP formulations is necessary to develop effective and safe LNP carrier formulations. By addressing the issue of reactogenicity, mRNA–LNP therapies hold promise for treating a wide range of non-infectious diseases, including those associated with chronic inflammation.

This review summarizes the current understanding of the reactogenicity of LNP carriers, including their potential to exacerbate underlying inflammation, impede the delivered mRNA translation, and potentially interfere with the repeated administration of mRNA therapeutics. Addressing a significant gap in the current literature regarding the reactogenicity of empty LNPs, our review seeks to bridge this critical void by providing a thorough and comprehensive analysis of the reactogenic manifestations associated with LNP carriers.

## 2. Exploring LNPs as Xenobiotics in Reactogenic Responses

Reactogenicity is a recognized phenomenon that manifests in individuals who are administered xenobiotics, which are defined as any foreign substances introduced into the body. This phenomenon encompasses a range of unfavorable events that may arise due to the immune system’s response to such substances, including mRNA–LNP formulations, which are presently employed in COVID-19 vaccines. These reactogenic outcomes in the form of adverse reactions stem from the immune system’s response to the LNPs themselves rather than the intended antigen of the vaccine. Although immunogenicity is an anticipated feature of LNP-based vaccines, the reactogenicity of mRNA–LNPs leads to undesirable therapeutic outcomes and unfavorable effects such as pain, fever, chills, and fatigue [4,9,22,23,24].

**Figure 1:**
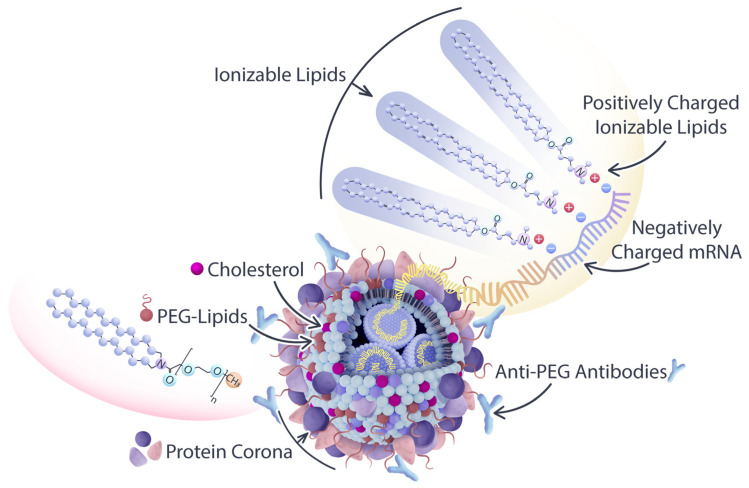
Composition of LNP carriers used in drug delivery: Their composition comprises ionizable lipids, helper lipids such as distearoylphosphatidylcholine (DSPC), cholesterol, and polyethylene glycol (PEG)-conjugated lipids. Among these components, ionizable lipids and PEG-lipids are the most reactogenic species, posing potential risks for unfavorable immune stimulation and adverse effects. PEG-lipids assume a significant role in LNP self-assembly, creating a hydrophilic steric barrier with protruding PEG chains surrounding the particle, while ionizable lipids bind to the nucleic acid payload through electrostatic interactions.

Studies are underway to evaluate the reactogenicity of LNPs and their structural components (Table 1) independently of the intended cargo. The typical composition of LNP carriers consists of ionizable lipids, helper lipids, such as distearoylphosphatidylcholine (DSPC), as well as cholesterol and PEG-lipids (Figure 1) [25]. Ionizable lipids are key components of LNPs, as they provide the positive charge that facilitates the encapsulation of negatively charged nucleic acids [26]. Helper lipids, such as DSPC, are often used to optimize the biophysical properties of LNPs. Helper lipids can enhance the stability of the LNP formulation and promote cellular uptake and endosomal escape of nanoparticles [27]. Cholesterol is another essential component of LNPs, as it also influences the biophysical properties of the LNP formulation. The presence of cholesterol within the LNP membrane can enhance its fluidity, thereby promoting the efficient release of nucleic acids from the LNP and facilitating their uptake by cells via enhanced fusogenicity [28]. Lastly, the incorporation of PEG-lipids into LNP formulations serves to augment their stability, decrease the activation of phagocytic immune cells, and enhance their pharmacokinetic properties [26]. In the process of LNP self-assembly, PEG-lipids assume a crucial role by creating a hydrophilic steric barrier through the formation of PEG chains on the LNP surface [29]. Van der Waals forces between the hydrocarbon chains of DSPE, PEG-lipids, and cholesterol molecules are essential for holding the LNPs together and maintaining their structural integrity [30]. The collective result is the formation of a PEG layer around the intact LNP, with the PEG chains protruding from the particle’s surface. This arrangement enhances the stability and integrity of the LNP structure, contributing to its overall functionality and effectiveness as a delivery vehicle. Hence, the incorporation of PEG-lipids to create a protective layer around the LNP serves to minimize its recognition by the immune system and enhances its in vivo circulation time. However, it is important to note that PEG-lipid incorporation can also lead to an increase in the immunogenicity of the formulation [31].

Among the components of LNP platforms, ionizable lipids and PEG-lipids are recognized as the most reactogenic elements [32]. Ionizable lipids such as Dlin-MC3-DMA (hereafter referred to as MC3), initially introduced in the therapeutic Onpattro (patirisan); SM-102, employed in the Moderna COVID-19 vaccine; and ALC-0315, utilized in the Pfizer-BioNTech COVID-19 vaccine are frequently evaluated and contrasted based on their reactogenicity and immunogenicity profiles, especially in the context of their implementation in vaccines (Table 1) [33,34,35]. Ionizable lipids are cationic at low pH and play a crucial role in encapsulating nucleic acids and promoting endosomal escape with the efficient release of cargo into the cytosol [34]. However, their positive charge can also interact with negatively charged endosomal membranes or proteins, resulting in cellular damage and undesired immune activation. Additionally, incorporating PEG-lipids into LNPs to enhance their stability, evade immune cell recognition, and impede opsonization by complement effector molecules can potentially lead to undesirable consequences. These may include the production of anti-PEG antibodies or the accumulation of PEG in various organs and tissues [31]. Importantly, the molecular weight of PEG was shown to play a crucial role in both the production of anti-PEG antibodies and their binding to the PEG moieties. A reduction in anti-PEG IgM formation was observed in mice when treated with LNPs conjugated to a fast-shedding PEG-lipid with short acyl chains, and hence lower molecular weight, in comparison with LNPs conjugated to a slow-shedding PEG-lipid with long acyl chains [36]. Moreover, the pretreatment of mice with slow-shedding PEG-containing LNPs resulted in the suboptimal performance of LNP formulations containing nucleic acids [36]. Interestingly, the administration of high-molecular-weight PEG as a pre-treatment in mice, before introducing PEGylated liposomal formulation, effectively sequestered the existing pool of anti-PEG antibodies [37]. The decrease in available anti-PEG antibodies enabled the prolonged circulation of PEGylated liposomes, avoiding the induction of accelerated clearance and preserving their efficacy. Additionally, PEG with a molecular weight of 2000 Da and larger exhibited the highest affinity for binding anti-PEG antibodies [38].

Therefore, it can be inferred that LNPs themselves, independently of their cargo, possess structural components capable of inducing reactogenic and immunogenic responses. However, evaluating the reactogenicity of individual components of LNP carriers (hereafter referred to as empty LNPs, eLNPs) in isolation is often challenging due to the integral role played by the assembled eLNPs in delivering nucleic acids and the hydrophobicity of separate eLNP components. As such, the comprehensive assessment of the safety and efficacy of the assembled product usually requires an evaluation of the reactogenicity of the intact eLNP platform independently of the intended cargo.

When conducting studies that include reactogenic profiling of mRNA–LNP formulations, researchers use multiple controls beyond the eLNP carriers. These include phosphate-buffered saline, placebo mRNA–LNPs incorporating luciferase or GFP mRNA, diluents, or naked nucleic acids [39,40,41,42,43,44]. Although LNPs are frequently employed as carriers for mRNA, utilizing eLNPs as a control for comparing their reactogenic effects to mRNA-loaded LNPs and attempting to separate their reactogenic profiles may not always yield optimal results. This is due to the possibility that eLNPs differ from mRNA-loaded LNPs in their size, composition, surface interactions, and fusibility potential. The molar ratio of PEG-lipids, the lipid-to-mRNA mass ratio, and the N/P ratio (representing the positively charged nitrogen of ionizable lipids and the negatively charged phosphates of nucleic acids) are critical factors that influence the ultimate composition of LNPs containing ionizable lipids for mRNA complexation [45]. eLNPs with an altered concentration of helper lipids and interference from ionizable lipids on the surface often display variations in their physicochemical and biological properties compared with mRNA-loaded LNPs, including a positive surface charge, increased splitting dynamics, and a modified protein corona composition [45]. Consequently, eLNPs may display different organ tropism as a result of their differential interaction with homing proteins, such as ApoE, compared with mRNA-loaded LNPs [45]. This, in turn, may augment the local and systemic eLNP reactogenicity.

Considering the major concerns surrounding the reactogenicity of eLNPs and their components, various efforts are underway to develop alternative lipid formulations that can minimize their adverse effects, while still maintaining the desirable properties of LNP platforms. It can be asserted with a high degree of confidence that, at present, the process of constructing LNPs entails adhering to comparable protocols with the primary variance arising from the application of different ionizable lipids (Table 1) but following similar LNP composition ratios (Table 2). For instance, the currently approved mRNA–LNP vaccines demonstrate a commonality in their structural compositions (Table 1). These compositions encompass a diverse range of ionizable lipids, each unique to the specific vaccine formulation, alongside the inclusion of diverse PEGylated lipids. Although the ionizable lipids utilized in each vaccine formulation differ, they all share certain structural features. These structural similarities, such as the amino-alcohol head group and branched hydrocarbon lipid tails characterized by ester linkages, contribute to the stability, mRNA encapsulation efficiency, and facilitation of cellular uptake of mRNA–LNP vaccines.

Despite the development of new formulations, the current Food and Drug Administration (FDA)-approved LNP platforms and their modified versions, which employ similar structural components, continue to serve as the established standard for delivering nucleic acids. Consequently, a comprehensive evaluation of their reactogenicity is imperative to expand the scope of their clinical applications. In light of this, it is essential to note that there are different types of reactogenicity assessments that can be used to evaluate the safety and efficacy of LNP-based therapies.

## 3. Assessment of Reactogenic Manifestations Following LNP Administration

**Figure 2:**
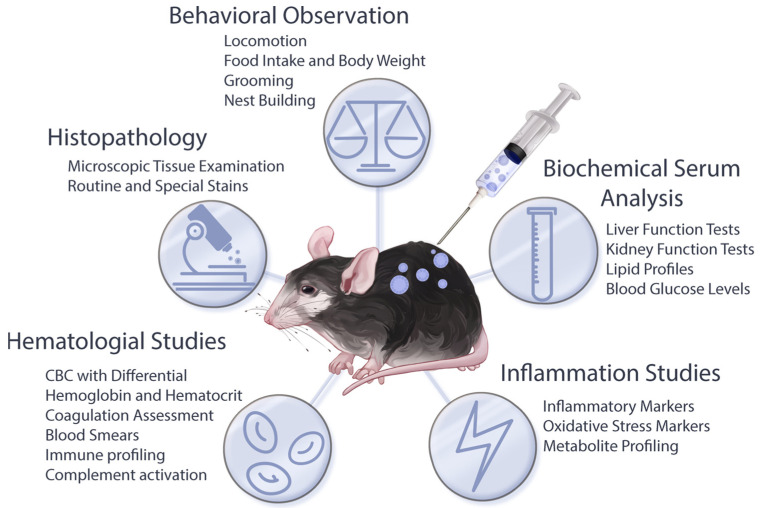
Methodological approaches for investigating reactogenic manifestations in animal models: By integrating different methodologies, such as behavioral observations, histopathological studies, and hematological, biochemical and inflammation analyses, a more comprehensive understanding of the cause-and-effect relationship between treatments and reactogenic manifestations can be achieved, aiding in the development of improved LNP platforms.

Evaluation of reactogenicity is a crucial component of preclinical safety studies for LNP formulations. Assessing reactogenicity can be carried out in several ways, depending on the administered formulation and the specific adverse events. While the assessment of reactogenicity in human subjects mainly involves evaluating the frequency and severity of local and systemic reactions, as well as monitoring for severe adverse events such as anaphylaxis, preclinical studies involving animal models typically employ a range of approaches. The employed methodologies in animal models encompass behavioral observations, hematological, immunological and biochemical analyses, histopathological examinations, and measurements of specific pro-inflammatory markers through immunohistochemistry and gene expression assays (Figure 2). The comprehensive utilization of these methods facilitates a more thorough understanding of the reactogenic manifestations associated with administered formulations.

*Behavioral observation* involves monitoring mice for signs of reactogenicity or toxicity, such as changes in food-motivated behavior, activity level, grooming, and respiratory distress. Food intake and body weight are essential parameters often closely examined in preclinical studies. Changes in these parameters provide valuable insights into the overall health of the animals and serve as early indicators of potential reactogenicity to xenobiotics. Therefore, in this review, food intake and body weight will be discussed further as important indicators of reactogenicity in mice.

The *hematological analysis* focuses on assessing blood samples for parameters such as platelet, white blood cell (WBC), and red blood cell (RBC) counts (i.e., complete blood count (CBC) with differential), hemoglobin concentration, and RBC characteristics. For instance, in rats and monkeys treated with MC3 eLNPs, no change was observed in RBC counts, hemoglobin, hematocrit, and RBC distribution width compared with PBS administration [49]. However, an increase in all these parameters was observed in response to mRNA-loaded LNPs. Additionally, rats exhibited a systemic increase in total WBC count resulting from mRNA-LNP treatment but not from MC3 eLNPs [49]. The systemic increase in WBCs found in that study mirrored the increasing mRNA concentration per administered dose. Interestingly, despite greater WBC counts in the mRNA-LNP treatment group, neutrophils and monocytes were increased in both the mRNA–LNP and MC3 eLNP treatment groups [49]. A transient increase in monocytes and neutrophils in the MC3 eLNP treatment group was observed for a shorter period (specifically, at day 9 post-administration), in comparison with mRNA–LNP treatment groups, where this increase was sustained (up to day 16 post-administration) [49].

In addition to CBC with differential, *immunological investigations*, including immune profiling and complement activation studies, play a crucial role in identifying specific components or characteristics of eLNPs that contribute to their reactogenic properties. Despite the absence of specific studies examining the activation of complement by eLNPs, ample evidence exists regarding complement activation through classical, mannose-binding lectin, and properdin-mediated pathways involving liposomes [57,58,59,60,61]. The activation of the complement cascade can occur through the recognition of structural components of liposomes or antibodies that target these components on the liposomal surface, including cholesterol, PEG, or structural lipids [62,63,64]. Additionally, the complement cascade can be initiated by the interactions of specific proteins that form the protein corona surrounding nanoparticles [61,65].

In addition to hematological and immunological assessment, blood samples for reactogenic studies can be evaluated for *biochemical parameters*, including liver enzymes, kidney function, and electrolyte balance. The most common biochemical approach utilized to assess systemic reactogenicity in animal models involves the evaluation of hepatic and renal function, as these organs are integral to the metabolism and elimination of foreign substances, including nanoparticles [66]. Exposure to reactogenic substances can result in liver or kidney damage, which is generally reflected by changes in the level of liver enzymes such as alanine and aspartate aminotransferases (AST, ALT) or by changes in markers of renal function, such as increased blood urea nitrogen (BUN) or plasma creatinine levels [67,68]. For instance, AST (but not ALT or alkaline phosphatase) was the only parameter significantly elevated in mice after MC3 eLNPs administration compared with placebo treatment [46]. In rats, the same type of eLNP containing MC3 ionizable lipids increased AST and ALT levels. Moreover, these parameters were increased to the same extent in the rats treated with the highest dose of the mRNA–LNP under investigation [49].

In addition to systemic manifestation, administration of eLNPs also yields local reactogenic reactions. Rodents typically manifest edema, redness, and infiltration of innate immune cells at the injection site. Additionally, innate immune cell infiltration is observed in organs penetrated by LNP-based formulations, such as the lung in studies with intranasal drug administration, mirroring similar responses observed in humans [23,49]. To evaluate the impact of xenobiotics on affected tissues, reactogenicity studies employ an assessment method involving *histopathological examination*, which entails the collection and microscopic analysis of tissue samples from various organs for signs of tissue damage, inflammation, or immune cell infiltration. For instance, histopathological assessment of liver samples showed that MC3 eLNPs induced individual cell necrosis in the livers of rats [49]. Similarly, histopathological approaches are employed to assess the infiltration of immune cells into organs and tissues. In mice, injection-site immune cell infiltrates consist of neutrophils, macrophages, and dendritic cells, and demonstrate the localized release of chemokines in response to eLNPs [23]. The intranasal delivery of labeled LNPs resulted in leukocytic infiltration of lung tissues, with a predominant presence of neutrophils and eosinophils [23]. Additionally, in rhesus macaques, injections with eLNPs and, to a greater extent, mRNA-loaded LNPs led to CD45-positive cell infiltration that denotes non-specific infiltration with hematopoietic cells [48]. Liang et al. also reported that neutrophil and monocyte infiltration in rhesus macaques was similar for mRNA-loaded LNP and eLNP treatments, regardless of administration route, when intramuscular and intradermal routes of injection were compared from immunogenic and reactogenic perspectives [48].

Finally, the assessment of pro-inflammatory effector molecules released in response to eLNP administration encompasses a range of *studies targeting inflammation* that include a wide array of methodologies, including ELISA, flow cytometry, cytokine profiling, quantitative PCR, and microarray analysis. Specifically, chemokine and cytokine expression studies can provide valuable insights into the reactogenicity pathways of eLNPs as a drug delivery system. For instance, the expression of chemokines at the injection site is known to facilitate the trafficking and recruitment of more circulating innate immune cells to the site of active inflammation [69]. Specifically, eLNPs induce the local release of multiple chemokines, including CCL2, CCL3, CCL4, CCL7, CCL12, CXCL1, and CXCL2, that further attract macrophages and monocytes [23]. The potent chemokine CCL2 is also overexpressed by monocytes in response to Acuitas eLNP administration in human peripheral blood mononuclear cells (PBMCs) ex vivo [50]. Collectively, eLNP carriers devoid of payload elicit the recruitment of monocytes and neutrophils, which subsequently amplify this cascade through the local expression of multiple effector molecules. However, eLNP administration elicits a response of shorter duration when contrasted with mRNA-loaded LNP treatments [49]. Our observation further suggests that this disparity may stem from the unique molecular-level responses to mRNA payloads and eLNP carriers, which can be recognized as danger- (DAMPs) or pathogen-associated molecular patterns (PAMPs).

## 4. Cellular and Molecular Responses to LNP Carriers

Within the intricate landscape of molecular interactions involving LNPs, innate immune cells emerge as the primary first responders to these therapeutics. Among these cellular “arenas”, macrophages and neutrophils take center stage as pivotal participants in the dynamic interplay with LNP-based therapies. Employing complex molecular processes, innate immune cells orchestrate a series of reactions that significantly impact the efficacy and safety of LNP formulations. Neutrophils play a critical role in the initiation of acute inflammation and are among the earliest immune cells to be recruited into tissues during such inflammatory processes [70]. Functioning as polymorphonuclear cells, they demonstrate remarkable versatility and contribute significantly to the innate immune response against invading pathogens and tissue damage [71]. Their rapid mobilization to inflamed sites is regulated by a diverse array of signaling molecules, including chemokines and cytokines, which guide their directed migration from the bloodstream to the sites of tissue inflammation [72,73,74]. Neutrophils interact with different nanoparticles, including LNPs and cationic liposomes [75,76,77,78]. Studies reveal that the surface characteristics of lipid-containing nanoparticles, such as PEG density and surface charge, play a pivotal role in modulating the neutrophils’ capacity to internalize LNPs [76,78]. Monocytes, the second most abundant cell type responding to LNPs, assume an important role in the context of tissue inflammation. This significance is attributed to their selective recruitment and guided migration into inflamed tissues, which is regulated by an array of inflammatory mediators encompassing cytokines, chemokines, and complement effector molecules [69]. Such an extravasation mechanism enables monocytes to actively participate in immune surveillance and host defense, as they engage with inflamed tissues to facilitate pathogen clearance, tissue repair, and the maintenance of tissue homeostasis [79,80]. Monocytes demonstrate proficient phagocytic capabilities and are equipped with pattern recognition receptors (PRRs), enabling them to actively interact with microbial pathogens, cellular debris, and diverse xenobiotics [81]. Macrophages are also widely recognized to be one of the initial and principal cell types responsible for nanoparticle processing [82]. As nanoparticles interact with the bloodstream, they become coated with opsonins, such as antibodies to PEG or complement effector molecules, which mark them for recognition by the mononuclear phagocytic system [82]. The mononuclear phagocytic system, including monocytes and macrophages, identifies opsonized nanoparticles, facilitating their efficient uptake and clearance from circulation [83,84,85]. This finely tuned process is essential for the immune surveillance and removal of foreign particles, contributing significantly to the body’s defense against potential threats posed by xenobiotics in the bloodstream.

Hematologic studies revealed that the administration of LNP formulations, including eLNPs, leads to a systemic increase in WBC levels. However, a more comprehensive understanding of the biological responses to these formulations can be gained by focusing on the innate immune cells’ reactions at the immediate site of administration. For instance, regardless of the eLNP administration mode, be it intravenous (IV), intramuscular (IM), intradermal (ID), or intranasal (IN), the cellular administration sites undergo infiltration by diverse innate immune cells. Among these cellular constituents, neutrophils, monocytes, macrophages, and, to a lesser extent, eosinophils and dendritic cells are identified as the predominant species in areas of infiltration [23,48,50]. This infiltration pattern highlights the robust and multifaceted response of the innate immune system to the presence of administered LNPs and signifies the importance of understanding the specific roles played by these immune cells. Furthermore, a localized approach that defines innate immune cell populations offers a comprehensive view of the early molecular events and interactions that shape the subsequent immune responses, thereby facilitating the development and optimization of LNP-based therapies.

**Figure 3:**
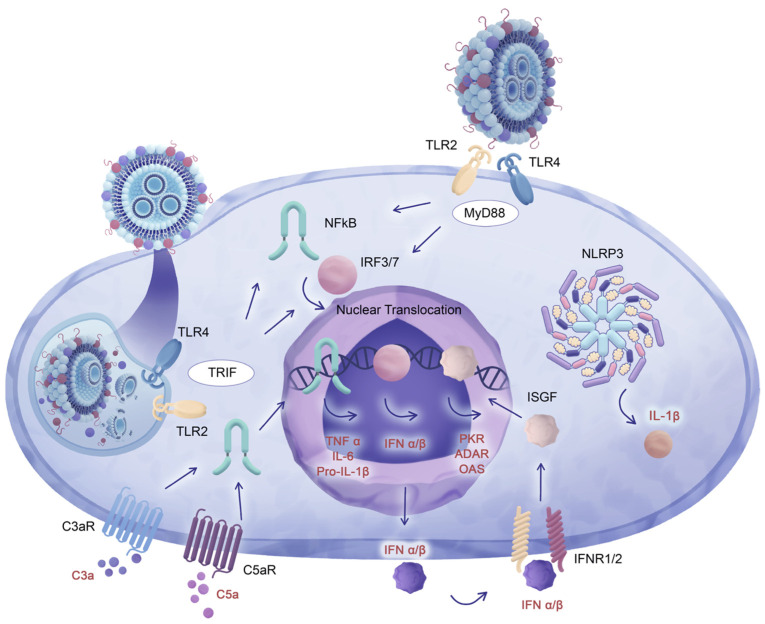
TLR4 signal propagation mediated by MyD88 and TRIF adaptor proteins: Converging pathways involving MyD88 and TRIF adaptors activate the cytokines TNF-α, IL-6, and IL-1β through nuclear translocation of the NF-κB, IRF3, and IRF7 transcription factors. Positive feedback loops and type I interferons enhance cytokine expression, activating additional pro-inflammatory effectors, and inducing cessation of mRNA expression. Complement cascade activation exacerbates cytokine-dependent reactogenicity, with C5a and C3a promoting inflammation via their receptors. TLR4 and C3aR/C5aR activation converge on MAPKs, increasing cytokine production by APCs.

The broad and diverse molecular pathways that underlie eLNP-related reactogenic manifestations as part of DAMP- and PAMP-mediated signaling involve the activation of toll-like receptors (TLRs), primarily TLR4 and TLR2 [50,86]. The interactions between eLNPs and TLRs are studied using downstream effectors and adaptor proteins that facilitate further signal transduction. Signal transduction from TLR4 and TLR2 necessitates the presence of the myeloid differentiation primary response 88 (MyD88) adaptor protein, which is essential for downstream signaling [87]. Additionally, some TLRs, such as TLR4, are known to modify their homophilic interactions with adjacent adaptor proteins based on the acidity of the local environment [87,88]. When TLR4 is internalized along with the forming endosome, the decrease in endosomal pH influences the exchange of the MyD88 adaptor protein for the TIR-domain-containing adaptor-inducing interferon-β (TRIF) adaptor protein [89]. It remains unclear which component of the LNP construct or its protein corona is responsible for TLR ligation, and it is also unknown whether the ligation occurs on the plasma membrane, where TLR4 associates with MyD88, or in the endosome, where TLR4 associates with TRIF for further signal transduction. Furthermore, TLR ligation can be facilitated in the endosome by both LNP surface structures and released LNP components (Figure 3). The acidification of the endosomal environment as endosomes undergo maturation induces the degradation of LNPs, releasing ionizable lipids from the LNP core [90,91]. In the case of LNP-mediated cargo delivery, this conversion occurs during the LNP processing within the endosomal compartment, followed by their escape from the endosomes to facilitate the release of their cargo into the cytosol. It is facilitated by the interactions between the surface of LNPs and the negatively charged membrane of the endosomes in addition to the decrease in ambient pH or by the action of helper lipids or cholesterol or its derivatives [92,93]. Apart from the destabilization of the endosomal membrane caused by LNPs, additional factors, such as the elevation of osmotic pressure or the swelling of particles within the endosome, can contribute to the facilitation of LNP disintegration and subsequent escape from the endosomes [91]. This process allows for the liberation of LNP contents into the cytosol, inevitably compromising the integrity of the intact particles.

Considering that TLR signaling, specifically TLR4-dependent signal propagation, can be categorized into two main pathways, namely MyD88- and TRIF- facilitated pathways (refer to Figure 3), it is crucial to employ both adaptor proteins in reactogenicity studies and to investigate their involvement in the eLNP-mediated induction of reactogenic manifestations. Therefore, considering the involvement of these proteins in propagating pro-inflammatory signals, researchers utilized genetic ablation approaches such as MyD88 and TRIF knock-out (KO) mouse models to investigate the impact of eLNPs on TLR receptors. For instance, MyD88 KO mice showed no response to empty LNPs in terms of follicular T-helper and germinal center B-cell upregulation, indicating that the adjuvant properties and, possibly, the reactogenicity of eLNPs rely on signal transduction via MyD88 adaptors [52]. Moreover, the convergence of signaling pathways dependent on MyD88 and TRIF adaptors results in the activation of a common set of cytokines, namely tumor necrosis factor-alpha (TNF-α), interleukin 6 (IL-6), and interleukin 1 β (IL-1β). This activation occurs through the induction of nuclear translocation of transcription factors such as nuclear factor-kappa B (NF-κB) and interferon regulatory factors 3 (IRF3) and 7 (IRF7). The expression of these cytokines is further enhanced by positive feedback loops involving type I interferons and interferon-stimulated gene factors (ISGFs) (Figure 3). Consequently, this amplification of cytokine expression leads to the activation of additional effectors, ultimately resulting in the global cessation of mRNA expression and the maturation of cytokines such as IL-1β that is associated with physiological reactogenic responses such as fever and painful sensations. Importantly, within the context of investigations concerning MyD88 and TRIF adaptors in LNP reactogenicity studies, mice with a dual knockout of MyD88 and TRIF exhibited reduced expression of downstream effectors, specifically TNF-α and IL-6, in comparison with wild-type (WT) mice when exposed to mRNA-loaded LNPs [94]. The study has a limitation from the perspective of reactogenicity investigations in that it did not distinguish between the effects of mRNA-loaded LNPs and empty LNPs on the MyD88/TRIF dual KO model. This is further complicated by the fact that both TRIF and MyD88 proteins also function as adaptors to TLRs that detect nucleic acids, such as TLR3, which recognizes double-stranded RNA (dsRNA) molecules as PAMPs and DAMPs [95]. Moreover, the MyD88 adaptor is involved in signal transduction from TLR7 that is activated by single-stranded RNA (ssRNA) [89]. As such, it is unclear whether the observed response is due to the nucleic acid payload or the LNP carrier or both. A further puzzle that arises in the decoding of reactogenicity of mRNA-loaded LNPs and eLNPs is that both the induction of TLRs by nucleic acids and eLNPs result in the expression of a similar subset of cytokines and chemokines involved in reactogenic symptoms. Additionally, multiple positive feedback loops and feeder pathways can exacerbate the expression of reactogenicity-related effectors.

Cytokine-dependent reactogenic manifestation can be exacerbated following complement cascade activation, where pro-inflammatory effects of complement effector molecules acting on their respective receptors result in the upregulation of IL-6, TNF-α, and IL-1β expression (Figure 3). The anaphylatoxins, C5a and C3a, are produced through proteolytic processes during the complement cascade activation, which involves the previously mentioned liposome-dependent activation of the classical, mannose-binding lectin, and properdin-mediated complement pathways [57,58,59,60,61,96]. The administration of LNPs possibly results in similar activation patterns, resulting in the generation of C3a and C5a effectors through the action of proteolytic convertases [97,98]. While functioning as anaphylatoxins, attracting innate and adaptive immune cells, and promoting the maturation of adaptive immune cells, C3a and C5a also interact with the C3a receptor (C3aR) and the C5a receptor (C5aR), propagating and amplifying the TLR-dependent inflammatory cascade [99,100,101,102,103,104,105]. For instance, the activation of TLR4 and C3aR/C5aR converges to the same mitogen-activated protein kinases (MAPKs), leading to the nuclear translocation of NF-κB, which results in a significant upregulation of IL-6, TNF-α, and IL-1β by antigen-presenting cells (APCs) [106,107].

## 5. Enhanced Cytokine Gene Expression in Response to eLNP Administration

**Figure 4:**
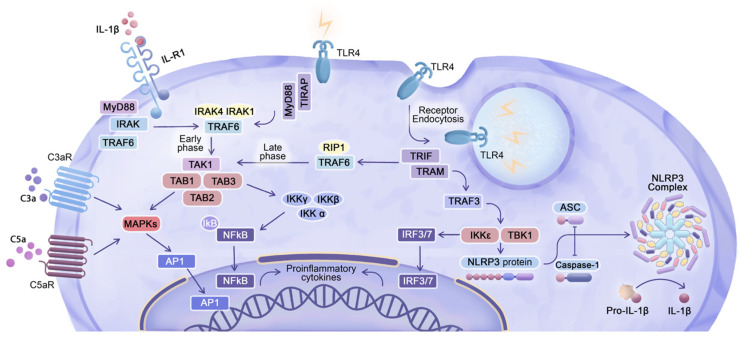
Integration of TLR4 signaling, inflammasome activation, and complement anaphylatoxin receptors in cytokine regulation: Upon TLR4 stimulation on the plasma membrane, a MyD88-dependent pathway is initiated, leading to the phosphorylation of interleukin-1 receptor-associated kinases (IRAKs), particularly IRAK1 and IRAK4. The recruited TNF-Receptor-Associated Factor 6 (TRAF6) undergoes ubiquitination, facilitating the formation of polyubiquitin chains that recruit transforming growth factor-β-activated kinase 1 (TAK1), TGF-beta-activated kinase 1 (MAP3K7), binding protein 1/2/3 (TAB 1/2/3), and inhibitor of nuclear factor-κB kinases (IKKs). These molecules are crucial for activating NF-κB and mitogen-activated protein kinase (MAPK) signaling. NF-κB translocates to the nucleus, acting as a transcription factor for pro-inflammatory cytokines such as TNF-α, IL-1β, and IL-6, signifying completion of the “early phase” of NF-κB activation. Activation of TLR4 in the endosome triggers a TRIF-dependent pathway, where translocating chain-associating membrane protein (TRAM) serves as an adaptor molecule connecting TLR4 and TRIF. TRAF6, recruited via TRIF, undergoes ubiquitination and interacts with receptor-interacting serine/threonine-protein kinase 1 (RIP1), facilitating the recruitment and activation of IKK for the “late phase” of NF-κB activation. Regardless of MyD88 or TRIF dependence, TLR activation induces NF-κB nuclear translocation, resulting in the expression of cytokines such as TNF-α, IL-6, IL-1β, and others, with temporal variations. Additionally, TNF-Receptor-Associated Factor 3 (TRAF3) interacts with TRIF and recruits TANK-binding kinase 1 protein (TBK1) and IκB kinase epsilon (IKKε) kinase, which phosphorylate and activate IRF3/7. IRF3/7 translocates to the nucleus, promoting the expression of type I interferons. Post-translational modifications are necessary to activate pro-IL-1β, with caspase-1 and apoptosis-associated speck-like protein containing CARD (ASC) adaptor activation by the NOD-, LRR-, and pyrin-domain-containing protein 3 (NLRP3) inflammasome complex playing a key role in this process. TRAF3, through IKKε and TBK1, can interact with NLRP3 inflammasome components in certain cellular contexts. Activated IL-1β, via interaction with IL-1R, can upregulate NF-κB nuclear translocation via a MyD88-dependent mechanism. Furthermore, C3aR and C5aR receptors ligated by C3a and C5a complement anaphylatoxins, lead to activation of the AP-1 and NF-κB transcription factors, either by interacting with MAPKs or IKKs, further propagating pro-inflammatory cytokine expression.

Reactogenic symptoms such as fever, chills, and localized inflammation at the injection site of the LNP-based vaccines are known to be driven by cytokine expression in the resident and migratory immune cells attracted to the injection site [108]. Recent research shed light on the mechanisms underlying these reactogenic manifestations, revealing that they are driven by certain cytokines, including IL-6, IL-1β, and TNF-α [109]. These cytokines are part of the body’s natural immune response and are responsible for inducing inflammation and fever, which help to mobilize the immune system to fight off the perceived target pathogen. The regulation of pro-inflammatory cytokine expression is tightly controlled by the previously mentioned transcription factors NF-κB, IRF3, and IRF7, which play crucial roles in the molecular mechanism of acute and chronic inflammation (Figure 4) [110,111].

The activation of transcription factors IRF3/7 is governed by the TRIF-dependent signaling via TLR4 upon ligation by eLNPs and via TLR3 upon ligation by dsRNA structures as integral components of secondary mRNA confirmations. [50,112,113]. The activation through the phosphorylation and subsequent nuclear translocation of IRF3/7 results in the expression of interferon β (IFN-β) and interferon-stimulated genes factors (ISGFs). Moreover, TLR stimulation with the subsequent MyD88-dependent signal transduction results in the expression and phosphorylation of another transcription factor, NF-κB, indicating the initiation of the “early” NF-κB pathway [114]. The phosphorylated NF-κB protein translocates to the nucleus, where it acts as a transcription factor to regulate the expression of pro-inflammatory effector molecules, including TNF-α, IL-1β, and IL-6 [110]. Together with IRF activation, the TRIF-dependent signaling pathway also guides the “late” activation of NF-κB, further emphasizing the convergence of TRIF- and MyD88-dependent pathways [114]. The converging nature of these pathways is elucidated in Figure 4. Overall, the pathway involving TLR activation leading to NF-κB nuclear translocation, regardless of whether it is MyD88- or TRIF-dependent, leads to the expression of cytokines such as TNF-α, IL-6, interleukin 8 (IL-8), IL-1β, and others, although the timing of their expression differs. In this context, the mechanism of transcriptional activation of IL-1β is somewhat complicated, because the interleukin-1 receptor (IL-1R) requires MyD88 as an adaptor molecule to transmit signals following IL-1R ligation by IL-1β (Figure 4) [115]. Therefore, the expression of *IL1β* in cells and tissues that encounter eLNPs presents the challenge of how the initial TLR-dependent expression of *IL1β* can be distinguished from its subsequent expression that is self-potentiated by IL-1β binding to IL-1R. Furthermore, at the protein level, post-translational modifications are necessary to activate the precursor of IL-1β, pro-IL-1β, and release the mature, biologically active form of IL-1β. Proteolytic activation of IL-1β is induced by the enzyme caspase-1 that is activated by the multi-protein complex inflammasome, NLRP3, which is assembled in response to TLR activation (Figure 4) [116]. Once activated, caspase-1 cleaves pro-IL-1β into its mature, active IL-1β form, which can then be secreted by the cell to exert its pro-inflammatory effects. In summary, the ligation of TLRs with the subsequent activation of different signaling pathways leads to the expression and post-translational activation of cytokines, including IFN-β, IL-6, TNF-α, and IL-1β, which are commonly used as outcome measures to study eLNP-induced reactogenicity.

The upregulation of pro-inflammatory cytokines in response to eLNP treatment has been studied in both ex vitro experiments and in vivo murine studies, with results showing increased expression over time and across different proprietary lipid formulations. eLNPs containing Acuitas’ proprietary lipids were assessed in ex vitro experiments with human PBMCs and in in vivo murine studies [50,52]. Expression of IL-1β and IL-6 was upregulated in monocytes and increased in a time-dependent manner after treatment with eLNPs [50]. Furthermore, Tahtinen et al. and Alameh et al. showed that the eLNPs containing SM-102 utilized in the Moderna COVID-19 vaccine and eLNPs containing the Alnylam ionizable lipid led to an increase in IL-1β levels in purified monocytes and draining lymph node lysate, respectively [52,53]. Levels of IL-1β and IL-6 were also increased in the sera of mice treated with eLNPs containing the MC3 and YK009 ionizable lipids compared with a control group treated with phosphate-buffered saline (PBS) [46]. Furthermore, serum TNF-α in this study was higher in mice treated with MC3 eLNPs than with mRNA-containing MC3 LNPs in the first 6 h post-treatment, although no information was provided about dosing considerations of eLNPs in comparison with mRNA–LNPs [46]. Interestingly, in contrast to TNF-α, levels of both IL-1β and IL-6 were increased in the eLNP treatment group, similarly to the mRNA-loaded LNP treatment group [46]. Parhiz et al. also found that MC3 eLNPs upregulated serum IL-6 in mice but were less reactogenic in terms of IL-6 levels compared with C12-200 ionizable-lipid-containing eLNPs [56]. In contrast, Tahtinen et al. reported that MC3 eLNPs did not change IL-1β levels; however, the doses of eLNPs administered to the mice were not reported in the study [53]. Sedic et al. found no change in IL-6 and TNF-α expression in rats subjected to MC3 eLNPs in comparison with the control group [49]. 

Despite the limited availability of data concerning eLNP reactogenicity in inflamed tissues, there is evidence indicating that pre-treatment with lipopolysaccharide (LPS), which is known to induce a TLR4-dependent innate immune response and acute inflammation, exacerbates existing inflammation following subsequent eLNP administration [56]. The exacerbation of inflammation induced by LPS may arise from various factors, such as heightened eLNP internalization or the self-potentiation of chemokine and cytokine expression via parallel or converging reactogenic pathways induced by LPS and eLNPs. Furthermore, LPS induces not only peripheral inflammation but also hypothalamic inflammation, thereby introducing a brain axis that leads to the induction of sickness behavior components, such as fatigue, fever, weight loss, decreased food intake, and anhedonia [117,118,119]. These findings emphasize the need to mitigate the reactogenic effects of LNPs to ensure their safe and effective application.

Abundant evidence from the studies demonstrates the utilization of not only diverse ionizable lipids but also different doses, emphasizing the significance of a comprehensive assessment of these factors to gain a more informed understanding of their impact on therapeutic applications. Table 2 showcases a selection of representative studies that vividly demonstrate the significant differences in eLNP doses employed. It emphasizes the wide range of doses utilized in the administered formulations. Therefore, the introduction of standardization in the doses and dosage regimens of eLNPs is crucial for facilitating a comparative evaluation of eLNP reactogenicity. Furthermore, standardization is particularly important for evaluating the safety profile of eLNPs to identify potential adverse reactions and to determine the optimal dose range for clinical use. Since pre-existing inflammation can increase the risk of adverse reactions to eLNPs, utilizing standardized dosing in research settings could help to identify the optimal dose range for minimizing the risk of exacerbating inflammation or of inducing further damage to cells and tissues.

## 6. LNP-Inducible Expression of Cytokines Modulating Sickness Behavior

**Figure 5:**
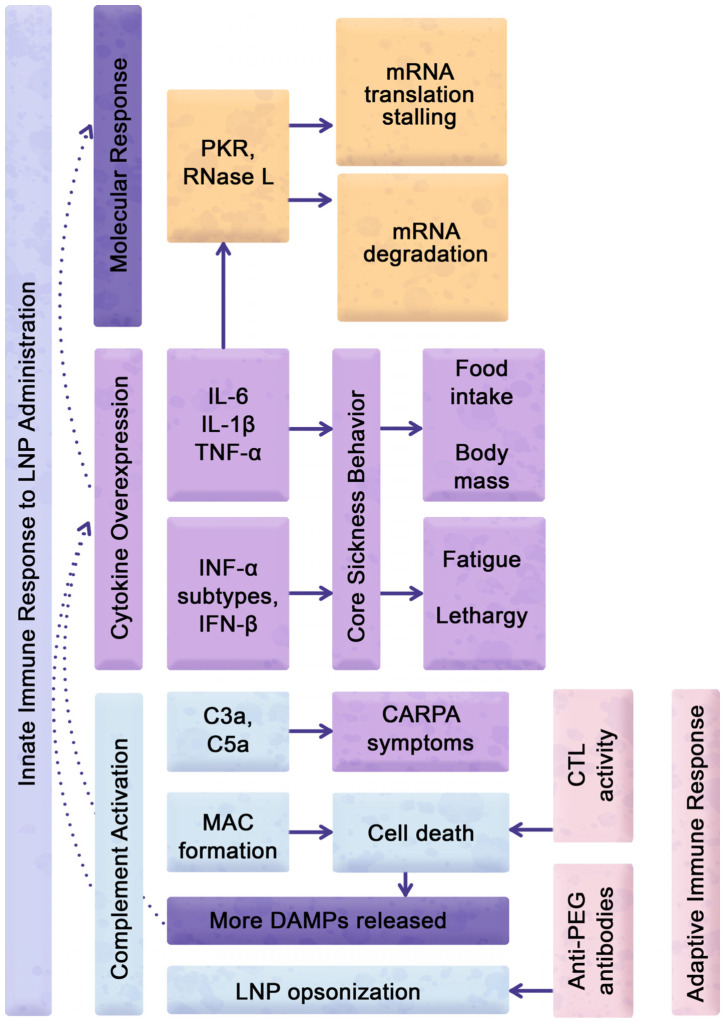
Mechanistic insights into reactogenic manifestations and therapeutic challenges in LNP administration: Complex mechanisms underlie reactogenic manifestations of LNP administration. Expression of cytokines, including IL-6, IL-1B, TNF-a, and interferons, initiate core sickness behaviors such as decreased food-motivated behaviors, fatigue, and lethargy. Concurrent LNP-dependent complement activation can result in CARPA symptoms, including anaphylaxis, chills, headache, and cardiopulmonary distress. Moreover, complement activation-dependent MAC formation potentially eliminates cells that are actively internalizing LNPs, leading to the release of additional DAMP signals and additional cytokine expression. CTLs also contribute to removing cells presenting LNP-related antigens from the treated cell pool. Complement opsonization of LNPs, along with anti-PEG antibodies, may increase systemic and local reactogenicity by sequestering particles and reduce their therapeutic efficacy. Additionally, activated PKR and RNase L enzymes can hinder mRNA translation or degrade the mRNA therapeutic payload, further decreasing the therapeutic efficacy.

The physiologic responses to eLNPs include a typical sickness response, consisting of attenuation of food intake and loss of body weight (Figure 5). This is driven, at least partially, by the action of pro-inflammatory cytokines on the hypothalamic centers regulating appetite and metabolism. Cytokines such as IL-6, IL-1β, and TNF-α play a role in the negative regulation of body weight in rodents as part of their sickness behavior in response to inflammatory conditions [120,121,122,123,124,125]. IL-6 is mainly expressed by innate immune cells, such as monocytes, but it is also produced by skeletal muscle and adipose tissues and has both pro- and anti-inflammatory effects [126,127,128,129]. In terms of body weight regulation, IL-6 exhibits a biphasic effect, where acute elevation of IL-6 levels decreases food intake and increases energy expenditure, while chronic elevation of IL-6 levels is associated with increased body weight and insulin resistance [123,130]. IL-1β is produced by a variety of cells, including innate immune cells such as macrophages, monocytes, and dendritic cells, and is known to be a potent pro-inflammatory cytokine [131,132]. Both IL-6 and IL-1β act on the hypothalamus, a key regulatory center in the brain that controls feeding behavior and energy expenditure [133]. IL-6 acts on the hypothalamus to increase the expression of anorexigenic (appetite-reducing) neuropeptides [134,135]. Although IL-1β increases the expression of orexigenic (appetite-stimulating) neuropeptides, such as neuropeptide Y (NPY) and agouti-related protein (AgRP), its potent induction of hypothalamic inflammation leads to a reduction in food intake and a consequent decrease in body weight [136,137].

The findings suggest that the elevated levels of IL-6 and IL-1β induced by eLNPs lead to decreased body weight through the modulation of hypothalamic function. IL-6 and IL-1β were shown to be increased at both the mRNA and protein levels in mice treated with eLNPs containing non-disclosed proprietary ionizable lipids [23]. Importantly, IL-6 and IL-1β overexpression was also associated with a decrease in murine body weight [23]. Of note, the authors also observed high mortality at the 10 μg eLNP dose delivered intranasally in this study, with just 20% survival on day 1 post-treatment [23]. Therefore, the observed weight reduction could be due to TLR-dependent septic shock involving a cytokine storm with the possible overexpression of IL-6, IL-1β, and TNF-α, as discussed in [138]. Importantly, the results of studies utilizing MyD88/TRIF KO mice that were administered mRNA–LNP injections suggest that MyD88/TRIF dual KO mice did not exhibit weight loss [94]. These findings suggest that effector molecules produced due to the LNP activation of TRIF- or MyD88-dependent reactogenic pathways play a pivotal role in inducing sickness behavior. Therefore, incorporating food intake and body weight as key indicators of systemic reactogenicity in the routine evaluation of eLNP is crucial, as they not only form an integral part of sickness behavior but are also paramount in assessing the efficacy and safety of LNP therapies for conditions that afflict patients’ frailty, such as cancer and other chronic diseases.

Together with a decrease in food-motivated behavior, fatigue is an important indicator of the severity of the reactogenic insult (Figure 5). Type I and Type II interferons play a critical role in developing disease-associated and iatrogenic fatigue syndromes [139,140,141,142,143,144]. Hence, the assessment of interferon expression holds significant importance due to their role in causing symptomatic reactogenic manifestations, including fatigue, in response to mRNA–LNP formulations. This is particularly relevant in the context of COVID-19 vaccine administration, where fatigue is a frequent side effect [145,146]. The role of interferons, along with other pro-inflammatory cytokines, is considered significant in both localized and systemic inflammation and in the development of fever [147]. Type I interferons, including IFN-α subtypes and IFN-β, are expressed as part of the pro-inflammatory cascade induced by unmodified mRNA acting on TLR3, TLR7, and TLR9 and by eLNPs acting on TLR2 or TLR4 [50,148,149]. Interferon-gamma (IFN-γ), or type II interferon, has lower pro-inflammatory potency and is important for the induction of adaptive immunity against intracellular pathogens [150]. Liang et al. reported that, in contrast to mRNA-loaded LNPs, eLNPs do not increase Type I IFN-inducible myxovirus resistance gene A (MxA) expression, which is used as a marker for the induction of endogenous type I interferons [48]. However, in the study by Connors et al., protein levels of IFNα and IFNγ were significantly increased after 24 h of Acuitas eLNP stimulation [50]. Connors et al. also report that eLNPs induced increased levels of phosphorylated IRF7, leading to the expression of ISGs [50]. Although there is a slight increase in IRF7 activation when TLR4 is ligated, significant activation only occurs when TLR7 or TLR9 are engaged, such as in the case of an LNP-encapsulated payload of nucleic acids acting on these receptors [149].

## 7. Reactogenicity Interference with Translation of mRNA Delivered by LNP Carriers

The immune response triggered by the injection of mRNA–LNP formulations can result in the downregulation of protein translation from the delivered mRNA, which limits the efficacy of mRNA-based therapies (Figure 5) [151,152]. The production of pro-inflammatory effector molecules downregulates expression of the delivered mRNA through various mechanisms, including the inhibition of translation initiation and the degradation of mRNA [153,154]. The immune system may recognize the eLNP carriers or eLNP structural components as a threat and mount an immune response, even in the absence of an mRNA payload. Significantly, compelling evidence suggests that mRNA–LNP formulations exhibit a substantial abundance of eLNPs that lack the incorporation of any therapeutic payload [45]. Hence, it is imperative to explore the reactogenic effects of eLNPs that may trigger immune responses and ultimately diminish mRNA expression in order to ensure optimal protein expression.

The delivery of mRNA using LNPs was observed to result in reduced mRNA translation when mice were simultaneously exposed to mild doses of LPS [152]. This finding holds significance as both eLNPs and LPS are capable of binding to TLR4. Lokugamage et al. further showed that administering a TLR4 inhibitor increased mRNA translation; however, it did not fully restore it to the levels observed in the absence of LPS treatment [152]. Furthermore, TLR4 activation by mRNA–LNPs themselves led to a decrease in mRNA translation. Importantly, the reduction in mRNA expression was not attributed to a decline in the particle endocytosis or endosomal escape of LNPs, but rather to the action of RNA-dependent protein kinase R (PKR) subsequent to TLR4 ligation. PKR, which reduces mRNA translation in the cell, is an intermediary component downstream of TLR signaling that, upon activation, phosphorylates eukaryotic initiation factor 2 (eIF2) [16,155,156]. This phosphorylation event prevents the recruitment of the initiator transfer RNA (tRNA) to the ribosome and the activation of eIF2 by binding to guanosine triphosphate (GTP), thereby inhibiting the initiation of translation [156,157,158,159]. Furthermore, one of the innate immune mechanisms by which effector molecules such as TNF-α, IL-1 family cytokines, type I IFNs, and other pro-inflammatory cytokines can inhibit translation initiation is by controlling the phosphorylation of the alpha subunit of eIF2, eIF2α [159,160]. Consequently, the initial event of TLR4 ligation, along with the subsequent expression of downstream molecules such as TNF-α, members of the IL-1 family, and type I interferons, contributes to the decreased expression of both therapeutic mRNA and the entire pool of mRNA within the cell as part of a reactogenic outcome.

In addition to inhibiting translation initiation, cytokines can also induce the degradation of mRNA molecules (Figure 5). One mechanism by which this occurs is through the activation of ribonuclease L (RNase L), an enzyme that degrades mRNA in response to exogenous nucleic acid insults, such as those arising from viral infection [161]. The activation of RNase L by pro-inflammatory cytokines can lead to both viral and host mRNA degradation, thereby reducing the overall level of mRNAs available for translation [162,163]. Moreover, pro-inflammatory cytokines can activate signaling pathways that lead to the induction of cell death, which also contributes to the downregulation of mRNA expression by the cells actively internalizing mRNA–LNP formulations [164]. Despite the low-reactogenicity modified mRNA formulations used in clinical practice, [17,18] the use of eLNP carriers may still cause reactogenic manifestations and subsequently lead to cytokine-dependent downregulation of mRNA expression.

The reduction in exogenous mRNA expression can be further modulated by activating complement and adaptive immunity by actively removing the cells internalizing mRNA–LNPs. Activation of the complement pathway by LNPs can initiate the formation of membrane attack complexes (MACs), which often inflict cellular damage. The formation of MACs as a result of activation through classical, mannose-binding lectin, and properdin-mediated complement pathways [165] not only poses a direct threat to the targeted cells but can also impact the uptake and internalization of LNP formulations, leading to a decrease in their therapeutic effectiveness. Additionally, anti-PEG antibodies and complement effector molecules play a critical role in the opsonization of LNPs, resulting in their accelerated clearance from the system and subsequently reducing their therapeutic efficacy. Furthermore, PEG incorporated into the LNP coating induces an anaphylactoid, complement-activation-related pseudoallergy (CARPA) rection [166,167]. CARPA is a harmful immune reaction triggered by the activation of the complement system, leading to adverse symptoms and potentially life-threatening complications in response to certain medications or therapeutic agents [166,168]. In their study, Ndeupen et al. put forth the hypothesis that PEGylated lipids present in LNPs may elicit CARPA in individuals who possess pre-existing PEG-specific antibodies. These antibodies could arise from the previous administration of mRNA–LNP formulations, including COVID-19 vaccines [23]. Of significant importance, extensive evidence exists regarding the activation of the adaptive immune system by LNP delivery, resulting in the generation of antibodies against immunogenic LNP components, including the formation of anti-PEG antibodies [20,169].

Furthermore, the activation of cytotoxic T lymphocyte (CTL) cells and their accumulation in draining lymph nodes adjacent to the xenobiotic injection site can lead to the destruction of cells such as APCs that have taken up LNPs containing the immunogenic components (Figure 5). Consequently, CTLs can facilitate an increase in the clearance of PEGylated LNPs, thereby causing a further decrease in the expression of the delivered mRNA. The actions of CTLs are indeed advantageous in therapeutic approaches that utilize mRNA–LNPs as cancer vaccines. The delivery of therapeutic mRNA–LNPs can lead to the expression of novel antigens in cancer cells that is potentiated by a triggered immune response [170]. However, this approach may be counterproductive in mRNA therapeutics aimed at applications beyond the elimination of diseased cells. These immune responses highlight the immunogenic potential of LNP bridging to reactogenic manifestations and emphasize the need for careful evaluation and monitoring of immune reactions in LNP-based therapeutic applications.

## 8. Reactogenicity Interference with Multiple Injections of Lipid Nanoparticle Formulations

Similar to the immune system’s ability to interfere with mRNA translation after a single injection, there is growing apprehension regarding the possibility of decreased mRNA expression in response to repeated administrations or after prior exposure to mRNA–LNP formulations. This phenomenon can be attributed to the recognition of the LNP delivery system as a foreign entity by the adaptive immune system, leading to an immediate immune response that may result in mRNA degradation prior to its translation into proteins. As the utilization of mRNA vaccines and therapeutics continues to expand, it is imperative to gain a comprehensive understanding of the potential long-term ramifications that may arise from repeated administrations. If repeated injections or pre-exposure to LNPs lead to a decrease in mRNA expression, it could impact the efficacy of these treatments and may require changes to their dosing, dosage, or delivery methods. Therefore, understanding the potential risks associated with repeated administration of mRNA–LNPs is important to improve the safety and efficacy of mRNA-based therapies.

Despite the limited amount of research on the pre-exposure to mRNA–LNP formulations decreasing mRNA expression, the available evidence suggests that there are significant implications for understanding its impact. In a study aiming to express an mRNA-encoded antigen, Qin et al. reported that pre-exposure to eLNPs or mRNA-loaded LNPs decreased the response in terms of both antibody production and germinal center B-cell expansion [54]. Further, upon administering mRNA–LNPs repeatedly, there was an observed transition in LNP distribution from the liver to the spleen after the third successive injection [171]. This observation was attributed to increased internalization of LNPs by splenic APCs, possibly recognizing the protein corona surrounding LNPs. Additionally, exposure of LNPs to plasma from LNP-immunized mice revealed the enhancement of immunoglobulins attached to the protein corona encircling the LNPs [171]. In line with prior research, the opsonizing immunoglobulins detected in this study included antibodies against PEG [171]. These findings suggest that the LNP carriers themselves, and not the mRNA payload, play a crucial role in the immune elimination of mRNA–LNP formulations. Of note, the physical characteristics of LNPs and their interactions with serum proteins can be altered by employing diverse ionizable lipid components, leading to variations in LNP accumulation across different tissues, such as liver, spleen, and lung [172]. Hence, the redistribution of LNPs following multiple injections may depend on the composition of the LNPs themselves.

In addition to stimulating adaptive immunity, pre-exposure to LNP formulations can also alter the innate immune response, which could aggravate mRNA elimination, decreasing its expression. For instance, the use of the BNT162b2 mRNA–LNP formulation as a vaccine resulted in the upregulation of pro-inflammatory and IFN-stimulated genes such as the chemokine CXCL10 and the cytokine IL-6 in monocytes [173]. These effects were more pronounced after the booster vaccination with BNT162b2 than after the initial vaccination [173]. Despite these findings, mRNA–LNP-based vaccines continue to play a crucial role in the ongoing battle against the COVID-19 pandemic, and their success provides a foundation for developing novel and innovative mRNA–LNP formulations for the treatment of various other diseases.

## 9. Conclusions and Next Steps in eLNP Reactogenicity Research

While LNP-mediated delivery of nucleic acid therapeutics offers substantial potential for disease prevention and treatment, optimizing their formulation to minimize side effects and reactogenicity is crucial. Furthermore, caution should be exercised when administering mRNA–LNP-based therapies to individuals with pre-existing chronic inflammation, as LNP reactogenicity may exacerbate the underlying inflammatory conditions. Additionally, even in healthy individuals, reactogenicity could pose a risk to the effectiveness of mRNA translation and may hinder the repeated administration of mRNA therapeutics. The current understanding of the effects of LNP composition, associated temporal kinetics, chronic dosage, and dosing on their ability to evoke reactogenic manifestations is still evolving, and further investigations are needed to develop safe and effective LNP carrier formulations.

Future studies should focus on elucidating the mechanisms of LNP-induced inflammation, as well as on identifying ways to mitigate their reactogenicity. For instance, temporal kinetics is an important consideration when studying eLNP reactogenicity, since the prolonged presence of LNP carriers can cause toxicity and interfere with the efficacy of the therapy. Therefore, the optimal timing and frequency of administration of LNP-based therapies must be carefully evaluated to minimize the risk of adverse reactions. The dose of LNP carriers and density of the mRNA payload distribution per particle is another factor that needs to be optimized for the desired therapeutic effect, especially in the setting of chronic administration. In addition, it is worth noting that variations in ionizable lipids, mRNA cargo, and PEG coating can significantly impact the reactogenicity of LNP carriers. Therefore, the standardization and optimization of LNP carrier formulations in reactogenicity studies can significantly enhance both their safety and effectiveness, paving the way for developing new treatments.

## Figures and Tables

**Table 1:** Key building blocks of representative FDA-approved mRNA–LNP formulations.

Category	Common Name	IUPAC Name	Chemical Structure
Ionizable Lipid	Dlin-MC3-DMA ^a^	(6Z,9Z,28Z,31Z)-6,9,28,31-Heptatriacontatetraen-19-yl 4-(dimethylamino)butanoate	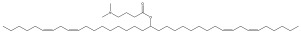
Ionizable Lipid	SM-102 ^b^	9-Heptadecanyl 8-{(2-hydroxyethyl)[6-oxo-6-(undecyloxy)hexyl]amino}octanoate	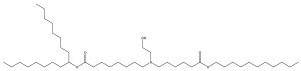
Ionizable Lipid	ALC-0315 ^c^	[(4-Hydroxybutyl)imino]di-6,1-hexanediyl bis(2-hexyldecanoate)	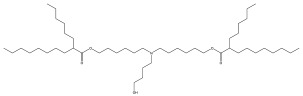
Helper Lipid	DSPC	(2R)-2,3-Bis(stearoyloxy)propyl 2-(trimethylammonio)ethyl phosphate	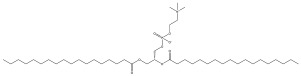
Stabilizing component	Cholesterol	(3β)-Cholest-5-en-3-ol	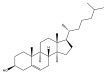
Shielding component	PEG	poly(oxyethylene)	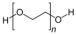

Ionizable lipids used in ^a^ Onpattro therapy, ^b^ Moderna, and ^c^ Pfizer-BioNTech COVID-19 vaccines.

**Table 2:** eLNP composition and doses utilized in reactogenicity studies.

Reference	eLNP Composition	Ionizable Lipid	eLNP Dose
[46]	IL: DSPC: cholesterol: PEG-lipid at a molar ratio of 50:10:38.5:1	MC3 and YK009	Not provided; mRNA–LNP dose was equivalent to 10 µg mRNA administered via the IM, SQ, or ID routes
[23]	IL: phosphatidylcholine: cholesterol: PEG-lipid at a molar ratio of 50:10:38.5:1.5 as described in [47]	IL under US10221127B2 patent (Acuitas Therapeutics)	10 μg administered in 4 spots, 2.5 μg/spot, ID, and IV; 10 μg administered IN
[48]	Valera LLC, a Moderna Therapeutics Venture, supplied all vaccines. In-house formulation: IL: DSPC: cholesterol: PEG-lipid: GLA at a molar ratio of 50:9.83:38.5:1.5:0.17	Not discussed	50 μg administered per site, injected ID
[49]	IL: DSPC: cholesterol: PEG-lipid at a molar ratio of 50:10:38.5:1.5	MC3 ionizable lipid	Equivalent to 0.3 mg/kg mRNA–LNP dose, injected IV
[50]	IL: DSPC: cholesterol: PEG-lipid at a molar ratio of 55:10:32.5:2.5 as described in [51]	IL under US10221127B2 patent (Acuitas Therapeutics)	Equivalent to 5 μg/mL total lipids or ~7.5 μg/mL ionizable lipids; eLNP used in in vitro study
[52]	The LNP formulation used in this study is proprietary to Acuitas Therapeutics	IL under US10221127 patent (Acuitas Therapeutics)	Total lipid content: 900 μg; equivalent to the lipid content of 30 μg mRNA-LNP ID and IV
[53]	IL: DSPC: cholesterol: PEG-lipid at a molar ratio of 50:20:28:2 for the MC3 or 50:10:38.5:1.5 for the SM-102 formulations	MC3 and SM-102	eLNP doses are not provided; eLNP injected IV
[54]	IL: phosphatidylcholine: cholesterol: PEG-lipid at a molar ratio of 50:10:38.5:1.5 mol/mol) as described in [47,55]	IL under US10221127 patent (Acuitas Therapeutics)	Equivalent to the lipid content of 2.5 μg mRNA-LNP injected ID
[56]	IL: DSPC: cholesterol: PEG-lipid at a molar ratio of 50:10:38.5:1.5	MC3 and C12-200	2 mg/kg lipids or dose equivalent to 0.32 mg mRNA/kg, injected IV

IL = ionizable lipid; Distearoylphosphatidylcholine = DSPC; ID = intradermally; IV = intravenously; IM = intramuscularly; SQ = subcutaneously; IN = intranasally.

## Data Availability

Data sharing is not applicable.

## Abbreviations

AP-1	Activator Protein 1
ASC	Apoptosis-Associated Speck-Like Protein Containing CARD
C3a	Complement Component 3a
C3aR	C3a Receptor
C5a	Complement Component 5a
C5aR	C5a Receptor
CARPA	Complement-Activation-Related Pseudoallergy
CTL	Cytotoxic T Lymphocytes
DAMPs	Danger-Associated Molecular Patterns
dsRNA	Double-stranded RNA
ELISA	Enzyme-Linked Immunosorbent Assay
eLNP	Empty Lipid Nanoparticle
eIF2	Eukaryotic Initiation Factor 2
FDA	Food and Drug Administration
GFP	Green Fluorescent Protein
IFN	Interferon
IL	Interleukin
IL-1β	Interleukin 1 β
IL-1R	Interleukin-1 Receptor
IL-6	Interleukin 6
IRAKs	Interleukin-1-Receptor-Associated Kinases
IRF	Interferon Regulatory Factor
KO	Knock-Out
LNP	Lipid Nanoparticle
MAC	Membrane Attack Complex
MAPKs	Mitogen-Activated Protein Kinases
MC3	Dlin-MC3-DMA
MyD88	Myeloid Differentiation Primary Response 88
N/P ratio	Nitrogen-to-phosphate ratio
NLRP3	NOD-, LRR-, and Pyrin Domain-Containing Protein 3
NF-κB	Nuclear Factor-Kappa B
PAMP	Pathogen-Associated Molecular Pattern
PBMCs	Peripheral Blood Mononuclear Cells
PEG	Polyethylene Glycol
PKR	Protein Kinase R
RBC	Red Blood Cell
TAK1	Transforming Growth Factor-β-Activated Kinase 1
TBK1	TANK-Binding Kinase 1
TLR	Toll-Like Receptor
TLR4	Toll-Like Receptor 4
TRAF6	TNF-Receptor-Associated Factor 6
TRAM	Translocating Chain-Associating Membrane Protein
TRIF	TIR-Domain-Containing Adaptor-Inducing Interferon-β
WT	Wild-Type
WBC	White Blood Cell
